# Erfahrungen mit der endoskopischen Ohrchirurgie an einer deutschen Hals‑, Nasen‑, Ohrenklinik der Maximalversorgung

**DOI:** 10.1007/s00106-023-01348-0

**Published:** 2023-08-20

**Authors:** Veronika Flockerzi, Bernhard Schick, Alessandro Bozzato

**Affiliations:** Klinik für Hals‑, Nasen- und Ohrenheilkunde, Universitätsklinikum des Saarlandes, 66421 Homburg, Deutschland

**Keywords:** Minimal-invasive Operationsverfahren, Endoskopie, Operationsdauer, Mittelohr, Cholesteatom, Minimally invasive surgical techniques, Endoscopy, Incision-suture-time, Middle ear, Cholesteatoma

## Abstract

**Hintergrund:**

Die vorliegende Arbeit berichtet über die Integration der endoskopischen Ohrchirurgie (EES) in den klinischen Alltag.

**Material und Methoden:**

In einer monozentrischen prospektiven Studie wurde über 10 Monate in geraden Wochen die Endoskopieeinheit zur Ohroperation mit aufgebaut und der Eingriff primär endoskopisch über einen transmeatalen Zugang begonnen. In ungeraden Wochen wurde auf das Endoskop verzichtet. Ausgewertet wurden 60 Eingriffe bei 59 PatientInnen. Vergleichspunkte waren die intraoperative Sicht, die Schnitt-Naht-Zeit, das postoperative Hörergebnis sowie der postoperative otoskopische Befund.

**Ergebnisse:**

Mit Ausnahme des Nervus facialis (*p* = 0,15 Mann-Whitney-U-Test) zeigte sich eine signifikant verbesserte Visualisierung aller Bereiche des Mittelohrs bei der EES. Die Schnitt-Naht-Zeiten waren im Methodenvergleich ähnlich. Sofern eine bimanuelle Platzierung von Ossikelprothesen notwendig war, verlängerte sich die Schnitt-Naht-Zeit überproportional (MES: 57,18 ± 9,7 min, EES: 76,83 ± 24,99 min; *p* = 0,019, signifikant da *p* < 0,05). Statistisch signifikante Änderungen bezogen auf die Hörergebnisse ergeben sich bei Vergleich der EES mit der mikroskopischen Technik nicht. In der Gruppe der EES-Operationen zeigten sich keine postoperativen Komplikationen.

**Schlussfolgerung:**

Die endoskopische Operationstechnik hat sich an einem realen Patientenkollektiv an unserem Standort bewährt.

## Abstract (en)

**Background:**

The aim of this article is to report on the integration of endoscopic ear surgery (EES) into daily clinical practice.

**Material and methods:**

In a monocentric prospective study, the endoscopy unit was set up during even weeks over a period of 10 months and the procedure was primarily started endoscopically via a transmeatal approach. In odd weeks, the endoscopy was omitted. A total of 60 procedures in 59 patients were evaluated. Points of comparison were intraoperative vision, incision-suture time, postoperative hearing outcome, and postoperative otoscopic findings.

**Results:**

With the exception of the facial nerve (*p* = 0.15 Mann-Whitney U‑test), the EES showed significantly improved visualization of all areas in the middle ear. The incision-suture times were similar in both methods. If bimanual placement of an ossicular prosthesis was necessary, the incision-suture time increased disproportionately (MES: 57.18 ± 9.7 min, EES: 76.83 ± 24.99 min; *p* = 0.019, *). There were no statistically significant changes related to hearing outcomes when comparing EES with the microscopic technique. There were no postoperative complications in the EES surgery group.

**Conclusion:**

Integration of EES proved to be successful and advantageous in a real patient collective at this location.

## Einleitung

In den vergangenen Jahren zeigte sich eine zunehmende Publikationsanzahl zum Thema „endoskopische Ohrchirurgie“, welche in der angloamerikanischen Literatur als „endoscopic ear surgery“ (EES) bezeichnet wird [4, 15]. Der im Vergleich zur verbreiteten mikroskopischen Technik mögliche minimal-invasivere Zugangsweg geht einher mit reduzierter Liegedauer, weniger Wundschmerzen und somit erhöhtem Patientenkomfort [11]. Dies trifft den aktuellen Zeitgeist, findet sich der behandelnde Arzt/die behandelnde Ärztin – nicht nur im universitären Setting – zwischen knappen zeitlichen Ressourcen bei gleichzeitig hohem Kostendruck und dem Anspruch nach minimal-invasiver Behandlung wieder [16].

In den Anfängen diente das Endoskop im Rahmen von Ohroperationen als additives Instrument um über die integrierte Winkeloptik Einsicht in weniger zugängliche Bereiche des Mittelohrs zu erhalten [14]. Aufgrund der endoskopischen Nasennebenhöhlenchirurgie ist das benötigte Instrumentarium vor Ort verfügbar und die OperateurInnen sind bereits mit der Handhabung vertraut.

Agha-Mir-Salim et al. [1] konnten nach einer Umfrage an 141 deutschen Universitäts- und Hauptabteilungen für HNO-Heilkunde, Kopf- und Halschirurgie von 45 Kliniken (32 %) Rückmeldungen hinsichtlich der aktiven Nutzung von endoskopischen Techniken in der Ohrchirurgie erhalten. Darunter gaben 27 Kliniken (60 % der beteiligten Kliniken) an, endoskopische Techniken meist ergänzend zur mikroskopischen Technik der Ohrchirurgie zu verwenden. Lediglich an einem Standort wurden alle Ohreingriffe ausschließlich endoskopisch durchgeführt. Nur 4 der 45 beteiligten Kliniken schätzten aber den zukünftigen Stellenwert der EES in Deutschland als „hoch“ ein – dies kontrastiert mit der Tatsache, dass allein 2021 annähernd 600 wissenschaftliche Artikel zum Thema EES publiziert wurden (https://pubmed.ncbi.nlm.nih.gov: Suchwort „endoscopic ear surgery“; „full text article“ vom 01.01.2021 bis 31.12.2021, Aufruf 06.01.2023).

Bisher wurden wenig deutschsprachige Arbeiten zum Thema veröffentlicht – zuletzt in einer Sonderausgabe der HNO [1]. Wir haben uns die Frage gestellt, wie die Einführung einer neuen Operationstechnik im klinischen Alltag gelingen kann.

## Methodik

**Figure 1:**
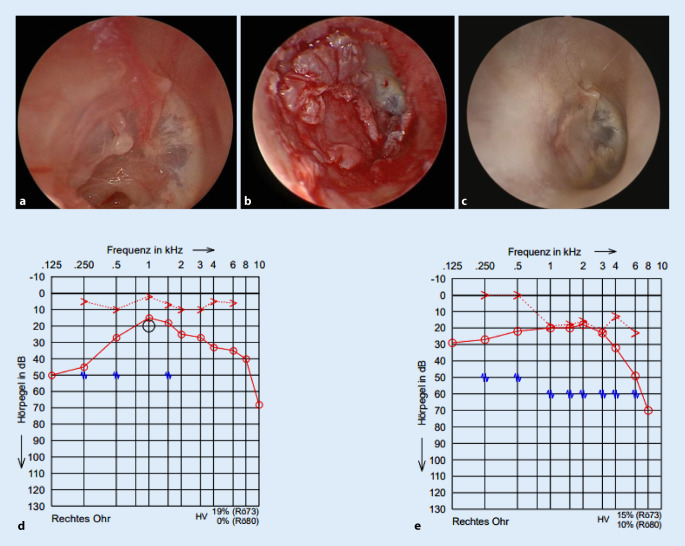

In unserer Klinik wird das volle Spektrum der Ohrchirurgie angeboten und es werden jährlich über 300 Ohreingriffe von 3 OhroperateurInnen durchgeführt. Mit dem Ziel der Einführung der endoskopischen Ohrchirurgie führten wir ab Februar 2017 monozentrisch prospektiv eine Datenerhebung durch. Eingeschlossen wurden Eingriffe eines Operateurs mit zum Studienbeginn 17 Jahren Erfahrung sowohl in mikroskopischer Ohrchirurgie als auch in endoskopischer Nasennebenhöhlenchirurgie. Von der Datenerhebung ausgeschlossen waren aufgrund der Subspezialisierung am Standort die Tumorchirurgie, die implantierbaren Hörsysteme, die Stapesplastik, Parazentese und Paukendrainage. Primär über einen retroaurikulären Zugang geplante Eingriffe wurden nicht in die Studie eingeschlossen. Der Studienzeitraum umfasste 10 Monate (01.02.2017 bis 31.10.2017). Das Vorgehen wurde abgestimmt mit der Ethikkommission bei der Ärztekammer des Saarlandes unter der Kennnummer 84/21. Vom Operateur wurden in Vorbereitung 2 Hospitationen durchgeführt und ein Workshop zum Thema „endoskopische Ohrchirurgie“ besucht. Um den klinischen Alltag möglichst realitätsnah abzubilden, wurde in geraden Wochen die Endoskopieeinheit zum jeweiligen vom Operateur durchgeführten Eingriff mit aufgebaut, in ungeraden Wochen wurde darauf verzichtet. Zusätzlich wurde ein endoskopisches Ohroperationssieb angeschafft. Jeder Eingriff in den geraden Wochen wurde primär endoskopisch über einen transmeatalen Zugang begonnen. Nach intraoperativem Bedarf konnte an beliebiger Stelle auf das vorgehaltene Mikroskop gewechselt und der Zugang erweitert werden. In den ungeraden Wochen wurden die Ohroperationen wie üblich mikroskopisch und überwiegend mittels endauralem Zugang durchgeführt. Nach dem endoskopischen Eingriff wurde ein Erhebungsbogen ausgefüllt. Hier waren die dokumentierten Endpunkte: i) Operationsdatum, ii) Diagnose, iii) durchgeführte Operation, iv) Schnitt-Naht-Zeit, v) Nutzung von Mikroskop/Endoskop gemäß Einteilung der Arbeitsgruppe von Cohen et al. ([4]; Tab. 1). Weiterhin wurde die Qualität der intraoperativen Sicht auf die anatomischen Areale im Mittelohr (rundes Fenster, ovales Fenster, Protympanon, Nervus facialis, Sinus tympani, Antrum, Epitympanon) erfasst, jeweils für den mikroskopischen als auch für den endoskopischen Blick unter Nutzung einer 5‑stufigen Likert-Skala. Hierbei war „5“ gleichbedeutend mit „exzellent“ und „1“ gleichbedeutend mit „sehr schlecht“. Die Operationsberichte wurden retrospektiv nach SAMEO-ATO^1^ [7] klassifiziert, um die chirurgische Vorgehensweise detailliert zu beschreiben (Tab. 2). Darüber hinaus wurden die prä- und postoperativen Hörergebnisse mittels Reintonaudiogramm verglichen, sowohl bezogen auf die Knochenleitungsschwelle als auch auf die Knochenleitung-Luftleitung-Differenz („air-bone gap“ [ABG]). Die präoperativen Reintonaudiogramme wurden bis zu 7 Tage präoperativ angefertigt, die postoperativen Messungen frühestens 8, spätestens 16 Wochen postoperativ. Die ABG wurde aus dem Mittelwert des Luftleitungsverlusts für die Frequenzen 0,5 Kilohertz (kHz), 1 kHz, 2 kHz und 4 kHz in dbHL („decibel hearing level“) bestimmt. Die Änderung der Knochenleitung-Luftleitung-Differenz wurden als ∆ABG, d. h. dem Verhältnis von präoperativem zu postoperativen ABG, dargestellt. Somit bedeutet ein negatives Vorzeichen eine Reduktion des ABG in dbHL und konsekutiv eine Verbesserung der gesamten Hörleistung (Abb. 1). Im Rahmen der Detamponade 3 Wochen postoperativ wurde ohrmikroskopisch der postoperative Trommelfellbefund erhoben.

**Table 1:** 

Kategorie	Beschreibung
0	Rein mikroskopische Operation
1	Endoskop zur Beurteilung, keine Präparation
2a	Präparation mit Mikroskop und Endoskop, > 50 % Nutzung Mikroskop
2b	Präparation mit Mikroskop und Endoskop, > 50 % Nutzung Endoskop
3	Rein endoskopische Operation

**Table 2:** 

SAMEO	EES	MES	ATO	EES	MES
S1	19	14	Ax	9	4
S2p	8	14	A1	13	17
S2r	3	2	A2	7	7
A1	10	0	A3	1	2
A2	0	0	Tx	6	1
A3	19	28	Tn	2	3
A4	1	2	T1	2	11
Mx	15	5	T2	20	14
M1a	1	0	T3	0	1
M1b	0	0	On	22	14
M2a	11	14	Ox	0	3
M2b	2	4	Osi	2	0
M2c	1	1	Osm	2	10
M3a	0	0	Ost	1	1
M3b	0	0	Osd	0	1
M1a + 2a	0	4	Ofi	0	0
M1b + 2a	0	2	Ofm	2	0
Ex	16	11	Oft	1	1
E1	14	19	Ofd	0	0
E2	0	0	Ovi	0	0
Ox	30	28	Ovm	0	0
O1	0	2	Ovt	0	0
O2	0	0	–		

*SAMEO-ATO* Akronym: Einteilung von Mastoidoperationen nach SAMEO: „stage of operation, approach, mastoidectomy procedure, external auditory canal reconstruction, obliteration of mastoid cavity“; Einteilung von Mittelohroperationen nach ATO: „access, tympanic membrane repair, ossicular chain repair“

Zur statistischen Analyse wurde der Shapiro-Wilk-Test zur Prüfung auf Normalverteilung durchgeführt. Da keine Normalverteilung vorlag, wurden Signifikanzen mittels Mann-Whitney-U-Test ermittelt. Das Signifikanzniveau wurde auf *p* < 0,05 festgelegt. Alle Berechnungen wurden durchgeführt unter Nutzung der GraphPad Prism® Software (Version 5.0, GraphPad Software, San Diego, CA, USA).

## Ergebnisse

### Studienkollektiv

Eingeschlossen wurden 60 Eingriffe bei 59 PatientInnen. In der Gruppe der mikroskopisch operierten PatientInnen erfolgten 30 Eingriffe bei 30 PatientInnen. Bei einem Patienten aus dem Kollektiv der endoskopisch Operierten wurden im erhobenen Zeitraum beide Ohren zeitversetzt operiert (30 Eingriffe bei 29 PatientInnen). Das Studienkollektiv der mikroskopischen Eingriffe (MES) umfasste 14 männliche und 16 weibliche PatientInnen ähnlich dem Studienkollektiv der endoskopischen Eingriffe (EES) mit 16 männlichen und 13 weiblichen PatientInnen. Die Verteilung der operierten Ohren war unterschiedlich: In der MES-Gruppe überwogen die rechtsseitigen Eingriffe mit 18 gegenüber 12 Eingriffen. In der EES-Gruppe wurde in 13 Fällen die rechte und in 17 Fällen die linke Seite operiert. Das Alter der PatientInnen der EES-Gruppe lag durchschnittlich bei 44,33 Jahren ± 22,6 Jahre (Altersspanne von 4 Jahren bis 82 Jahren). Das Alter der PatientInnen der MES-Guppe lag vergleichbar durchschnittlich bei 41,87 Jahren ± 19,6 Jahren (Altersspanne 6–77 Jahre). Zur Übersicht wurde die heterogen zusammengesetzte Gruppe der verschiedenen Eingriffe gemäß SAMEO-ATO (Tab. 2) thematisch weiter zusammengefasst in: i) Myringoplastiken, ii) Tympanomeatoplastiken bei Otitis media mesotympanalis, iii) Eingriffe bei Cholesteatom/Adhäsivprozess, iv) Tympanoskopie und v) Gehörgangsoperation. Die Verteilung der Eingriffe ist in Tab. 3 dargestellt.

**Table 3:** 

	MES-Gruppe		EES-Gruppe			
	Ersteingriff	Revision	Ersteingriff		Revision	
Gesamt	18	12	20		10	
Myringoplastik	3	–	4	Typ 3: 4x	–	
Tympanomeatoplastik bei Otitis media mesotympanalis	6	5	7	Typ 2a: 1x Typ 2b: 4x Typ 3: 2x	5	Typ 2a: 3x Typ 2b: 1x Typ 3: 1x
Eingriffe bei Cholesteatom/Adhäsivprozess	5	7	4	Typ 2a: 4x	5	Typ 2a: 4x Typ 3: 1x
Tympanoskopie	1	–	3	Typ 3: 3x	–	
Gehörgangseingriff	3	–	2	Typ 2b: 2x	–	

*MES* „microscopic ear surgery“, *EES* „endoscopic ear surgery“

Im Kollektiv der EES wurde in 7 Eingriffen eine Ossikelprothese implantiert (Tab. 2: Osi = 1, Osm = 2, Ost = 1, Ofm = 2, Oft = 1), davon waren 3 Eingriffe eine Tympanoplastik Typ IIIA und 3 Eingriffe eine Tympanoplastik Typ IIIB, ein Eingriff eine Tympanoplastik Typ II nach Wullstein [20]. 5 dieser Eingriffe wurden bei Otitis media mesotympanalis notwendig. Die übrigen beiden Eingriffe waren aufgrund von Cholesteatom oder Adhäsivprozess erforderlich. Im Kollektiv der MES wurden 4 Tympanoplastiken Typ IIIA und 4 Tympanoplastiken Typ IIIB durchgeführt. Nur 2 dieser Operationen wurden aufgrund einer chronischen Otitis media mesotympanalis notwendig, die übrigen Eingriffe waren begründet durch Cholesteatome oder Adhäsivprozesse.

### Nutzung von Mikroskop und Endoskop

Unter dem Aspekt Nutzung Mikroskop versus Endoskop wurde 12-mal eine Operation Cohen Typ 2a, 7‑mal eine Operation Cohen Typ 2b und 11-mal eine Operation Cohen Typ 3, d. h. ein rein endoskopischer Eingriff durchgeführt [4]. Unter diesen letztgenannten Eingriffen waren 3 Tympanoskopien (Indikation: 2‑mal akute Surditas, einmal unklare Schallleitungsschwerhörigkeit) und 4 Myringoplastiken bei traumatischer Trommelfellperforation. Auffällig war zudem, dass sofern in der EES-Gruppe das Platzieren einer Ossikelprothese (im Rahmen der 7 Tympanoplastiken Typ II und III, s. oben) notwendig war, über die Hälfte der Zeit mikroskopisch operiert wurde im Sinne einer Operation Cohen Typ 2a.

### Qualität der intraoperativen Sicht

**Figure 2:**
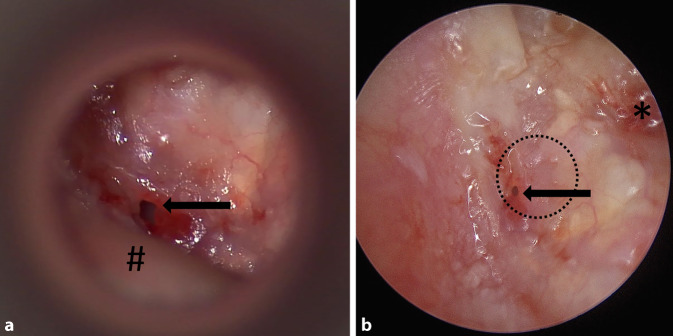

Bei Einzelbetrachtung der jeweiligen anatomischen Areale zeigte sich mit Ausnahme des Nervus facialis eine signifikant bessere Visualisierung aller Bereiche des Mittelohrs mittels Endoskop, für den Sinus tympani und das Protympanon und das Trommelfell war dies hochsignifikant (Tab. 4, Abb. 2).

**Table 4:** 

	Endoskop	Mikroskop	*p*-Wert
Trommelfell	4,7 ± 0,6	3,9 ± 0,31	*p* < 0,0001*
Rundes Fenster	4,77 ± 0,51	4,11 ± 0,57	*p* = 0,0002*
Ovales Fenster	4,64 ± 0,56	4,05 ± 0,69	*p* = 0,0026*
Protympanon	4,89 ± 0,31	2,94 ± 0,94	*p* < 0,0001*
Nervus facialis	4,17 ± 0,80	3,79 ± 0,85	*p* = 0,15
Sinus tympani	4,60 ± 0,51	2,8 ± 1,01	*p* < 0,0001*
Antrum	3,81 ± 0,83	3,11 ± 0,81	*p* = 0,0108*
Epitympanon	3,89 ± 0,85	3,26 ± 0,73	*p* = 0,0194*

*Asterix* Signifikanzniveau erreicht

### Schnitt-Naht-Zeit

**Figure 3:**
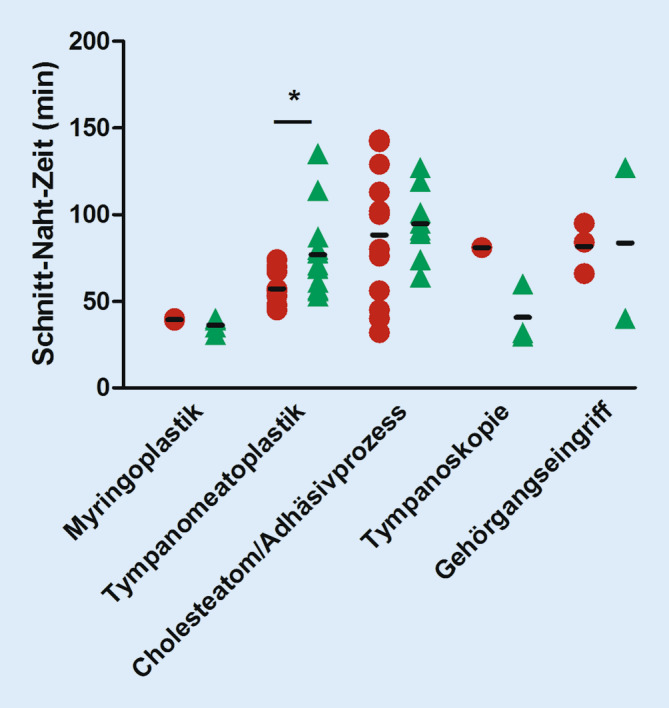

Aufgrund der heterogenen Gruppe der durchgeführten Operationen stellte sich eine vergleichende Gegenüberstellung endoskopischer und mikroskopischer Eingriffe und dem Gesichtspunkt der Schnitt-Naht-Zeit als schwierig dar (Abb. 3). Statistisch sinnvoll war eine vergleichende Gegenüberstellung der Schnitt-Naht-Zeit in der Gruppe der Tympanomeatoplastiken bei chronischer Otitis media mesotympanalis sowie den Cholesteatomen/Adhäsivprozessen. Für letztgenannte Gruppe fand sich keine signifikante Verlängerung der Eingriffszeit in der EES-Gruppe. Signifikant verlängert war die Schnitt-Naht-Zeit für die EES-Gruppe bezogen auf Tympanomeatoplastiken bei chronischer Otitis media mesotympanalis. Für Myringoplastiken (MES: 39,33 ± 0,57 min, EES: 36,25 ± 4,11 min), Tympanoskopien (MES: 81 min, EES: 40,66 ± 16,77 min) und Gehörgangseingriffe (MES: 81,67 ± 14,64 min, EES: 83,5 ± 61,5 min) waren die mittleren Schnitt-Naht-Zeiten nicht vergleichbar.

### Hörergebnisse

**Figure 4:**
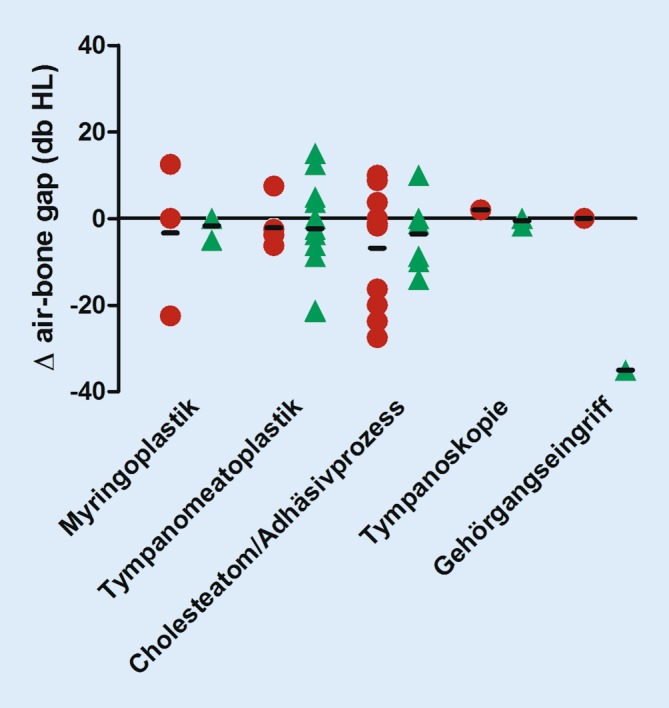

Während bei allen PatientInnen präoperative Reintonaudiogramme vorlagen, erschienen aus der Kohorte der EES-PatientInnen insgesamt 4 nicht zur postoperativen Hörkontrolle versus 8 in der Kohorte der MES-PatientInnen. Die Werte der Knochenleitungskurve blieben sowohl in der EES-Gruppe als auch in der MES-Gruppe postoperativ gegenüber präoperativ statistisch unverändert. In der MES-Gruppe war die postoperative mittlere Knochenleitung-Luftleitung-Differenz in 73 % gleich oder besser als präoperativ, in der EES-Gruppe entsprechend 81 %. Statistisch signifikante Änderungen des ABG bezüglich der prä- und postoperativen Werte (∆ABG, gemittelt bei 0,5, 1, 2 und 4 kHz in dbHL) ergaben sich nicht (Abb. 4).

### Otoskopischer Befund bei Detamponade

Regelgerecht erfolgte die Detamponade nach hausinternem Schema 3 Wochen postoperativ. Bei einem der operierten Ohren in der MES-Gruppe lag eine residuelle Trommelfellperforation über einen halben Quadranten vor. Daher wurde eine Revisionsoperation zum Trommelfellverschluss geplant, die Daten dieses Eingriffs gingen nicht in die Studie ein. In der EES-Gruppe wurden keine Trommelfelldefekte beobachtet. In keinem Fall kam es zu einer Infektion im Wundbereich des operativen Zugangswegs.

## Diskussion

Als Limitation der Studie ist die geringe Zahl durchgeführter Myringoplastiken, Tympanoskopien und Gehörgangseingriffen zu nennen, die keine tiefergehende statistische Analyse zuließen. Ziel der vorliegenden Arbeit war es im Sinne einer Methodenetablierung die Abläufe im Rahmen der EES zu optimieren und die Technik in den klinischen Alltag zu integrieren.

Insbesondere die Visualisierung der Mittelohranatomie und -pathologie gelingt, wie dargelegt, signifikant besser als mit dem mikroskopischen Blick alleine. Dieser Aspekt findet sich in der internationalen Literatur, teils aus subjektivem Eindruck der Operateure, teils aus quantifizierter Analyse wieder [5, 13, 19]. Ein weiterer Vorteil liegt gemäß Preyer [16] darin, dass im Kontext der operativen Ausbildung künftiger OhrchirurgInnen sowohl OperateurIn als auch AssistentInnen oder fortgeschrittenere ChirurgInnen denselben Blick auf den Operationssitus haben. Anatomische Kenntnisse können so einfacher vermittelt und auch einem größeren Publikum gleichermaßen zugänglich gemacht werden. Auch die anfängliche Skepsis seitens Operationspflege (Mehrarbeit durch Aufbau von Endoskopieeinheit *und* Videoturm zum Mikroskop) wich nach wenigen Eingriffen der Begeisterung, da erstmals auch der instrumentierenden Operationspflege eine Einsicht in das Operationspflege und somit die Operationsschritte begreifbarer wurden. So konnte die zu Beginn beim Aufbau investierte Zeit durch vorausschauendes Instrumentieren mehr als ausgeglichen werden.

In der Literatur [12, 18] finden sich unterschiedliche Angaben, wie viele Eingriffe benötigt werden, um sicher im Umgang mit der endoskopischen Technik zu werden. Die Zahlen reichen von 30–100, wobei insbesondere die ersten 5 Eingriffe häufig signifikant längere Schnitt-Naht-Zeiten haben. Wir bemerkten einen deutlich routinierteren Gesamtablauf nach dem zweiten Monat des Beobachtungszeitraums. In der vorliegenden Arbeit fand sich eine signifikante Verlängerung der Schnitt-Naht-Zeit für die endoskopisch durchgeführten Tympanomeatoplastiken bei chronischer Otitis media mesotympanalis gegenüber dem mikroskopischen Vorgehen, wohingegen alle übrigen Eingriffe vergleichbare Schnitt-Naht-Zeiten hatten. Wir erklären uns diesen Aspekt mit dem relativen Überwiegen von Tympanoplastiken Typ II und III in dieser Gruppe. Beim endoskopischen Vorgehen war das gewohnte bimanuelle Platzieren der Prothesen nicht immer möglich, es musste mitunter auf das bereitgestellte Mikroskop gewechselt werden. Der Wechsel auf das Mikroskop machte in 5 der 7 eingegangenen Fälle zusätzliche Bohrarbeiten wie das Absenken der hinteren Gehörgangswand notwendig, um die notwendige Sicht in das Mittelohr zu erlangen. Konsekutiv kam es zu einer Verlängerung der Schnitt-Naht-Zeit. Nichtsdestotrotz konnte die Arbeitsgruppen um Das et al. zeigen, dass nach entsprechender Übung keine Unterschiede in der Schnitt-Naht-Zeit auch für Tympanoplastiken Typ II und III bestehen [6]. Eine retrospektive Analyse der Schnitt-Naht-Zeiten endoskopischer und mikroskopischer Ohroperationen aus dem Jahr 2021 [17] bei der ein dem unseren ähnliches Patientenkollektiv betrachtet wurde, zeigte ebenfalls keine Nachteile der endoskopischen Technik bezogen auf die Schnitt-Naht-Zeit. Zwischenzeitlich ist unser Eindruck, dass nach annähernd 5 Jahren regelmäßig genutzter endoskopischer Technik das einhändige Platzieren der Prothesen kein zeitintensiver Faktor mehr ist, in Summe aber mehr als 30 Eingriffe notwendig waren, um die Geschicklichkeit dahingehend zu trainieren.

Hinreichend belegt und in unserem Kollektiv nachvollziehbar ist, dass sich je nach genutzter Methode keine Änderungen ergeben bezogen auf die postoperative Hörleistung [3, 8, 9, 17, 21].

Wir entschieden uns in der vorliegenden Arbeit bewusst für ein inhomogenes Patientenkollektiv, um den realen operativen Alltag am Standort abzubilden. Konsekutiv wurden auch Kinder mit in die Studiengruppe eingeschlossen. Die postoperativen Ergebnisse sind gemäß Literatur unabhängig von mikroskopischer oder endoskopischer Technik beginnend bei Kindern unter 3 Jahren vergleichbar [2, 3, 10]. Ein Aspekt, der die Nutzung von Endoskopen bei Kleinkindern vereinfacht, ist der verhältnismäßig kurze und gerade ausgeformte Gehörgang.

## Fazit für die Praxis

- Die endoskopische Operationstechnik hat sich an einem realen Patientenkollektiv an unserem Standort bewährt.
- Die Visualisierung der Mittelohranatomie ist mittels des Endoskops signifikant besser als mit dem Mikroskop alleine.
- Sowohl OperateurIn als auch ZuschauerIn haben bei der endoskopischen Ohrchirurgie (EES) denselben Blick auf den Operationssitus. Anatomische Kenntnisse können einfach vermittelt und weitergegeben werden.
- Bei der EES ergeben sich keine Änderungen bezogen auf die postoperative Hörleistung. Der Zugangsweg ist weniger traumatisch.
- Auch Kinder können in endoskopischer Technik operiert werden.

## Footnotes

1 Akronym: Einteilung von Mastoidoperationen nach SAMEO: „stage of operation, approach, mastoidectomy procedure, external auditory canal reconstruction, obliteration of mastoid cavity“; Einteilung von Mittelohroperationen nach ATO: „access, tympanic membrane repair, ossicular chain repair“.
